# Beneficial Effects of Long-Term Administration of an Oral Formulation of Angiotensin-(1–7) in Infarcted Rats

**DOI:** 10.1155/2012/795452

**Published:** 2012-02-09

**Authors:** Fúlvia D. Marques, Marcos B. Melo, Leandro E. Souza, Maria Claúdia C. Irigoyen, Rúben D. Sinisterra, Frederico B. de Sousa, Sílvia Q. Savergnini, Vinícius B. A. Braga, Anderson J. Ferreira, Robson A. S. Santos

**Affiliations:** ^1^National Institute of Science and Technology in Nanobiopharmaceutics, Federal University of Minas Gerais, 31.270-901 Belo Horizonte, MG, Brazil; ^2^Department of Physiology and Biophysics, Federal University of Minas Gerais, 31.270-901 Belo Horizonte, MG, Brazil; ^3^Department of Chemistry, Federal University of Minas Gerais, 31.270-901 Belo Horizonte, MG, Brazil; ^4^Department of Morphology, Federal University of Minas Gerais, 31.270-901 Belo Horizonte, MG, Brazil

## Abstract

In this study was evaluated the chronic cardiac effects of a formulation developed by including angiotensin(Ang)-(1–7) in hydroxypropyl **β**-cyclodextrin (HP**β**CD), in infarcted rats. Myocardial infarction (MI) was induced by left coronary artery occlusion. HP**β**CD/Ang-(1–7) was administered for 60 days (76 **μ**g/Kg/once a day/gavage) starting immediately before infarction. Echocardiography was utilized to evaluate usual cardiac parameters, and radial strain method was used to analyze the velocity and displacement of myocardial fibers at initial time and 15, 30, and 50 days after surgery. Real-time PCR was utilized to evaluate the fibrotic signaling involved in the remodeling process. Once-a-day oral HP**β**CD/Ang-(1–7) administration improved the cardiac function and reduced the deleterious effects induced by MI on TGF-**β** and collagen type I expression, as well as on the velocity and displacement of myocardial fibers. These findings confirm cardioprotective effects of Ang-(1–7) and indicate HP**β**CD/Ang-(1–7) as a feasible formulation for long-term oral administration of this heptapeptide.

## 1. Introduction

Cardiovascular diseases remain the leading cause of morbidity and mortality worldwide mainly due to their ischemic conditions [1]. Moreover, coronary artery disease is the most common reason of heart failure in Westernized nations [2]. Thus, the continuous search for therapies that are effective in reducing the incidence of these pathologies is still imperative.

Since its discovery in 1988 [3], the biologically active heptapeptide angiotensin(Ang)-(1–7) has been widely studied. This is mainly due to the observation that its effects are often opposite to those attributed to Ang II, whose actions favor the development of pathologic conditions in the heart [2, 4] and in other organs [5, 6] by binding to the AT_1_ receptor. In fact, several studies have demonstrated that Ang-(1–7) exerts beneficial effects in various organs [7, 8], including the heart. In this organ, it promotes antiarrhythmogenic effects [9], potentiation of the bradykinin vasodilatory effect [10], improvement of the cardiac function [11–15], reduction of the release of norepinephrine [16], and regulation of the cell growth and cardiac remodeling [4, 17–20]. Many of these effects are mediated by the activation of the Mas receptor [4, 9, 10, 14, 18] which was identified as an endogenous binding site for Ang-(1–7) [21].

In the heart, an increased activity of the classical axis of the renin-angiotensin system (RAS) composed by angiotensin-converting enzyme (ACE), Ang II, and AT_1_ receptor leads to ventricular hypertrophy, heart failure [22, 23], and fibrosis [4, 19]. The excessive fibrosis caused by maladaptive remodeling processes contributes to the diastolic and systolic dysfunction by increasing the myocardial stiffness and by reducing the pumping capacity [24]. The locally produced cytokine transforming growth factor *β* (TGF-*β*) is a major mediator of this process [25]. Its expression is increased in many cardiac pathologies such as hypertrophic and dilated cardiomyopathy [26–28] and myocardial infarction [29, 30]. In this latter condition, evidences suggest that TGF-*β* has a central role in the inflammatory and fibrotic phase of the healing process and may critically modulate many cellular steps of the postinfarction repair process by mediating cardiomyocyte growth, fibroblast activation, and extracellular matrix deposition [30]. Furthermore, TGF-*β* is considered an important marker for the transition course of stable hypertrophy to heart failure [31].

Recently, we have demonstrated that the inclusion of Ang-(1–7) into the oligosaccharide hydroxypropyl *β*-cyclodextrin (HP*β*CD) [32] is an effective formulation for oral administration of this heptapeptide [12]. Here, we aimed to evaluate the effects of long-term administration of the HP*β*CD/Ang-(1–7) inclusion compound on cardiac dysfunction and fibrosis caused by myocardial infarction (MI) in rats. Additionally, the expression levels of collagen type I and TGF-*β* were also analyzed in the hearts. 

## 2. Methods

### 2.1. Animals

Male Wistar rats weighing 180 to 210 g (approximately 3 months of age) were used in this study. The animals were provided by the animal facilities of the Biological Sciences Institute (CEBIO, Federal University of Minas Gerais) and housed in a temperature- and humidity-controlled room maintained on a 12:12-h light-dark schedule with free access to food and water. All animal procedures were performed in accordance with institutional guidelines approved by local authorities. 

### 2.2. Experimental Groups

The animals were divided into three groups: sham surgery treated with HP*β*CD (*n* = 8), vehicle-treated MI (infarction plus HP*β*CD, *n* = 7), and MI + HP*β*CD/Ang-(1–7) [infarction plus HP*β*CD/Ang-(1–7), *n* = 7]. The treatment with vehicle (HP*β*CD; 46 *μ*g/kg/day in distilled water by gavage) or HP*β*CD/Ang-(1–7) (76 *μ*g/kg/day in distilled water by gavage) started in the first day of MI, and the rats were killed, and the hearts were harvested for real-time PCR analysis 60 days after the beginning of the treatment. The final volume of gavage [HP*β*CD and HP*β*CD/Ang-(1–7)] was approximately 0.5 mL. Thirty-one animals initiated the experimental protocols. Three rats died within 48 hours after the MI surgery and one sham-operated animal died after one week. Additionally, five animals were excluded due to abnormal increases in the right atria detected by the echocardiographic exam at the initial examination or due to marked weight loss during the period of treatment.

### 2.3. Myocardial Infarction

MI was induced by proximal left anterior descending (LAD) coronary artery occlusion and performed under anesthesia with 10% ketamine/2% xylazine (4 : 3, 0.1 mL/100 g, i.p.). The animals were placed in supine position on a surgical table, tracheotomized, intubated, and ventilated with room air using a respirator for small rodents. The chest was opened by a left thoracotomy at the third or fourth intercostal space. After the incision of the pericardium, the heart was quickly removed from the thoracic cavity and moved to the left to allow access to the proximal LAD coronary artery. A 4-0 silk suture was snared around the LAD and carefully ligated to occlude the vessel. The heart was then placed back, and the chest was closed with 4-0 silk sutures. Sham-operated rats were treated in the same manner, but the coronary artery was not ligated.

### 2.4. Echocardiography Analysis

Animals underwent transthoracic echocardiographic examination before the surgery and after 15, 30, and 50 days of LAD coronary artery ligation. *In vivo* cardiac morphology and function were assessed noninvasively using a high-frequency, high-resolution echocardiographic system consisting of a VEVO 2100 ultrasound machine equipped with a 16–21 MHz bifrequencial transducer (Visual Sonics, Toronto, Canada). The rats were anaesthetized with 3.5% isoflurane for induction, the anterior chest was shaved, and the rats were placed in supine position on an imaging stage equipped with built-in electrocardiographic electrodes for continuous heart rate monitoring and a heater to maintain the body temperature at 37°C. Anesthesia was sustained via a nose cone with 2.5% isoflurane. High-resolution images were obtained in the right and left parasternal long and short axes and apical orientations. Standard B-mode images of the heart and pulsed Doppler images of the mitral and tricuspid inflow were acquired. Left ventricular (LV) dimensions and wall thickness were measured at the level of the papillary muscles in left and right parasternal short axis during the end systole and end diastole. LV ejection fraction (EF), fractional shortening (FS), and mass were measured. All the measurements and calculations were done in accordance with the American Society of Echocardiography. The following M-mode measurements were performed: LV internal dimensions at diastole and systole (LVIDD and LVIDS, resp.), LV posterior wall dimensions at diastole and systole (LVPWD and LVPWS, resp.), and interventricular septal dimensions at diastole and systole (IVSDD and IVSDS, resp.). Based on these parameters, end diastolic and end systolic LV volumes (EDLVV and ESLVV, resp.), FS, EF, stroke volume (SV), and cardiac output (CO) were calculated. Also, the radial strain from the bidimensional long axis view of the left ventricle was performed using the Vevostrain software. The following parameters were evaluated: velocity, displacement, strain, and strain rate. 

### 2.5. Plasma Ang-(1–7) Levels Measurement

Blood samples were collected in tubes through a polypropylene funnel after the decapitation of the animals. These tubes contained 1 mmol/L *p*-hydroxymercuribenzoate, 30 mmol/L 1,10-phenanthroline, 1 mmol/L PMSF, 1 mmol/L pepstatin A, and 7.5% EDTA (50 *μ*L/mL of blood). After centrifugation, plasma samples were frozen in dry ice and stored at −80°C. Peptides were extracted onto a BondElut phenylsilane cartridge (Varian). The columns were preactivated by sequential washes with 10 mL of 99.9% acetonitrile/0.1% heptafluorobutyric acid (HFBA), and 10 mL of 0.1% HFBA. After sample application, the columns were washed with 20 mL of 0.1% HFBA and 3 mL of 20% acetonitrile/0.1% HFBA. The adsorbed peptides were eluted with 3 mL of 99.9% acetonitrile/0.1% HFBA into polypropylene tubes rinsed with 0.1% fat-free BSA. After evaporation, the Ang-(1–7) levels were measured by radioimmunoassay (RIA), as previously described [33].

### 2.6. Reverse Transcription and Real-Time PCR

To perform the real-time PCR analysis, the hearts were cut transversally approximately 1 mm below the suture point and 3 mm above the apex; thereby, they were divided in 3 parts: basal, middle, and apical. Only the middle portion, where the majority of the infarcted tissue was localized, was used for real-time PCR analysis. Total mRNA isolation was performed following the TRIzol reagent protocol (Invitrogen Life Technologies). Seven hundred nanograms of mRNA treated with DNAse were used as template for M-MLV reverse transcriptase (ArrayScript, Ambion) using the following antisense primers: Mas (3′-GGTGGAGAAAAGCAAGGAGA-5′), TGF-*β* (3′-GGTTCATGTCATGGATGGTGC-5′), collagen I (3′-CCTTAGGCCATTGTGTATGC-5′), and S26 (3′-CGTGCTTCCCAAGCTCTATGT-5′). Real-time PCR was carried out immediately after the synthesis of the first strand cDNA. The sense primers used were Mas (5′-ACTGTCGGGCGGTCATCATC-3′), TGF-*β* (5′-TGACGTCACTGGAGTTGTACGG-3′), collagen I (5′-TGTTCAGCTTTGTGGACCTC-3′), and S26 (5′-CGATTCCTGACAACCTTGCTATG-3′) and their respective antisense primers as mentioned above. The PCR reactions containing 300 *μ*M of each primer (sense and antisense), 50–100 ng of cDNA, and SYBR Green PCR Master Mix (Applied Biosystems) were run under standard conditions in an ABI Prism 7000 Sequence Detector. The threshold cycle (CT) was determined for each sample, and the CT values of the S26 were subtracted from the CT values of the experimental samples to obtain ΔCT values. Transcript levels in left ventricles were expressed as fold relative to the S26 values (2-ΔΔCT).

### 2.7. Statistical Analysis

All data are expressed as means ± SEM. Echocardiographic data were estimated using two-way ANOVA followed by the Bonferroni posttest. The real-time PCR data, variation of velocity, and displacement of each time in relation to the initial time were analyzed using one-way ANOVA followed by the Newman-Keuls posttest. The level of significance was set at *P* < 0.05 (GraphPad Prism 4.0).

## 3. Results

### 3.1. Plasma Ang-(1–7) Levels

Plasma levels of Ang-(1–7) in blood samples collected 24 hours after the last dose of HP*β*CD/Ang-(1–7) in MI rats were higher than those observed in vehicle-treated rats; however, the difference did not reach significant statistical difference (80.16 ± 18.4 pg/mL versus 49.7 ± 13.6 pg/mL). Plasma levels of Ang-(1–7) in vehicle-treated sham rats averaged 63 ± 11.7 pg/mL.

### 3.2. Effects of HP*β*CD/Ang-(1–7) Long-Term Administration on Echocardiographic Parameters

**Figure 1:**
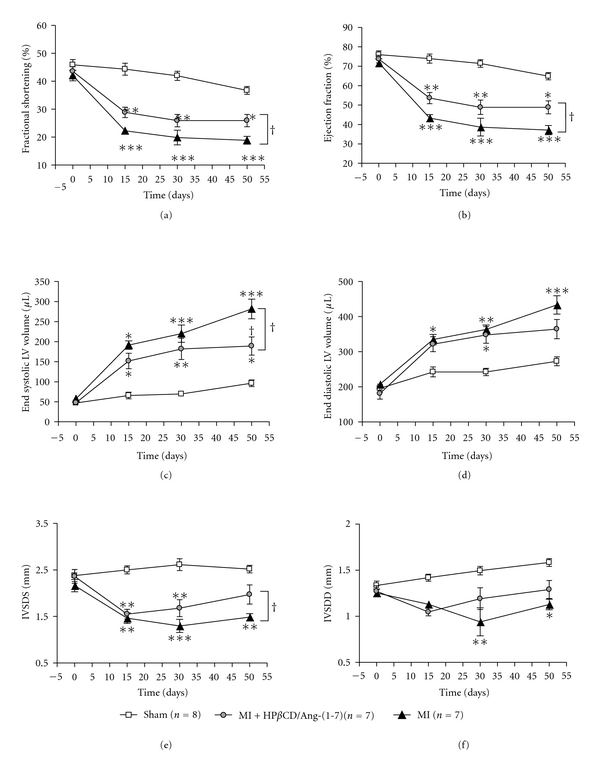
Effects of HP*β*CD/Ang-(1–7) on echocardiographic parameters after left coronary artery ligation in rats followed for up to 50 days. (a) Fractional shortening, (b) ejection fraction, (c) end systolic left ventricular volume, (d) end diastolic left ventricular volume, (e) interventricular septal dimension in systole, and (f) interventricular septal dimension in diastole. *P < 0.05  versus sham; **P < 0.01  versus sham; ***P < 0.001 versus sham; ^†^ *P* < 0.05 versus MI (two-way ANOVA followed by the Bonferroni posttest).

**Figure 2:**
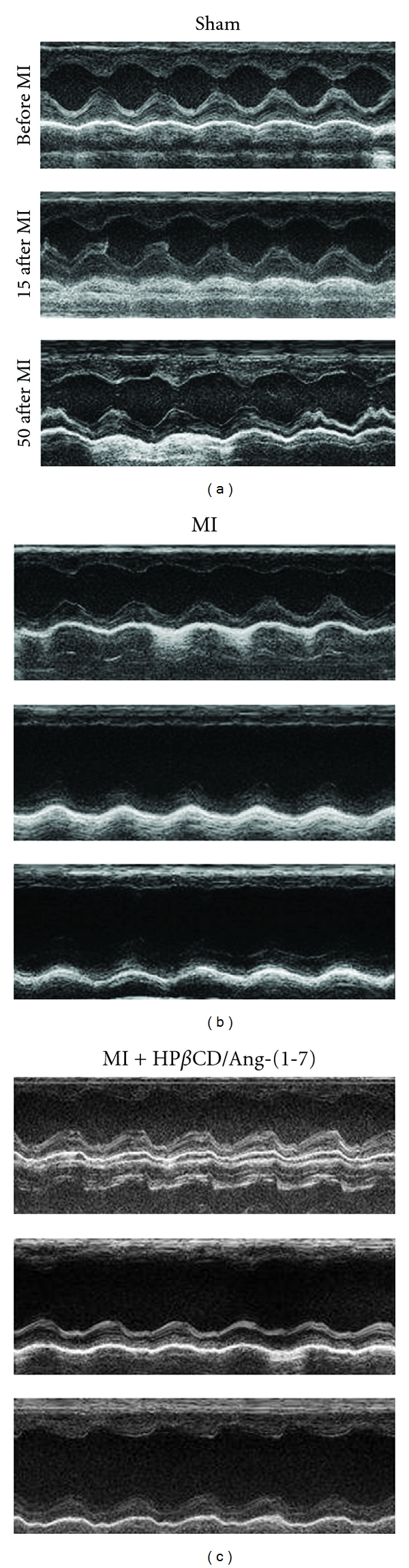
Representative M-mode images showing cardiac function and left ventricle chamber dimensions in sham, MI, and MI + HP*β*CD/Ang-(1–7)-treated rats. Note the marked increase in end-systolic dimension (ESD) and in end-diastolic dimension (EDD) after infarction and the improvement in the cardiac function after 50 days in rats treated with HP*β*CD/Ang-(1–7).

The echocardiographic analysis at the initial time (before the surgery) showed that SV, HR, CO, EF, FS, EDLVV and ESLVV, LV mass, IVSDD, and IVSDS were similar in all three groups evaluated (data not shown). The success of the MI procedure was confirmed by the presence of one of the following changes in the myocardial kinetics observed during the echocardiographic analysis: (i) hypokinesia caused by reduction in the thickness or wall motion, (ii) akinesia represented by absence of thickening and/or movement, and (iii) dyskinesia characterized by changes in the movement in one or more segments or regions of the heart. Although the body weight gain was less pronounced in MI vehicle-treated animals, no significant differences were detected among the groups during the treatment period (Table 1). Also, no significant changes were observed in the LV mass, SV, HR, and CO during the treatment period. However, the administration of the inclusion compound to infarcted animals allowed them to better recover the SV and CO over time (Table 1). As expected, MI caused a progressive impairment of the cardiac function evidenced by decreases in the FS (Figure 1(a)), EF (Figure 1(b)), IVSDS (Figure 1(e)), and IVSDD (Figure 1(f)) and increases in the ESLVV (Figure 1(c)) and EDLVV (Figure 1(d)). The administration of HP*β*CD/Ang-(1–7) significantly improved the FS, EF, IVSDS, and ESLVV of MI animals (Figure 1). Specifically, after 15 days of surgical procedure, MI induced a significant reduction in the FS (50%), EF (42%), IVSDS (44%), and a significant increase in the EDLVV (40%) and ESLVV (189%) (Figure 1). The HP*β*CD/Ang-(1–7) treatment ameliorated the decrease of FS, EF, and ESLVV; that is, FS increased 32% (29 ± 2% versus 22 ± 1% in vehicle-treated MI rats), EF increased 26% (54 ± 3% versus 43 ± 2% in vehicle-treated MI rats), and ESLVV decreased 20% (152 ± 19 *μ*L versus 191 ± 10 *μ*L in vehicle-treated MI rats). Thirty days after MI induction, control infarcted animals showed a similar profile as observed at the 15 days of MI. An increase of 52% and 214% in the EDLVV and ESLVV was observed, respectively. Furthermore, an additional reduction in the IVSDS of untreated MI rats was observed (36%). Again, the treatment with HP*β*CD/Ang-(1–7) induced an improvement in all parameters analyzed, including a significant attenuation of the reduction in the IVSDD (1.2 ± 0.11 mm versus 0.9 ± 0.13 mm in vehicle-treated MI rats). At the end of the 50 days postinfarction period, no further alterations in the cardiac function of the vehicle-treated infarcted rats were observed; in contrast, the HP*β*CD/Ang-(1–7)-treated rats showed an improvement of all cardiac parameters evaluated, that is, FS (26 ± 2% versus 19 ± 1% in vehicle-treated MI rats), EF (49 ± 3% versus 37 ± 2% in vehicle-treated MI rats), IVSDS (2.0 ± 0.21 mm versus 1.5 ± 0.08 mm in vehicle-treated MI rats), IVSDD (1.3 ± 0.1 mm versus 1.1 ± 0.06 mm in vehicle-treated MI rats), EDLVV (364 ± 27 *μ*L versus 445 ± 27 *μ*L in vehicle-treated MI rats), and ESLVV (189 ± 23 *μ*L versus 282 ± 25 *μ*L in vehicle-treated MI rats) (Figures 1 and 2).

### 3.3. Effects of Long-Term Administration of HP*β*CD/Ang-(1–7) on Radial Strain Parameters

**Figure 3:**
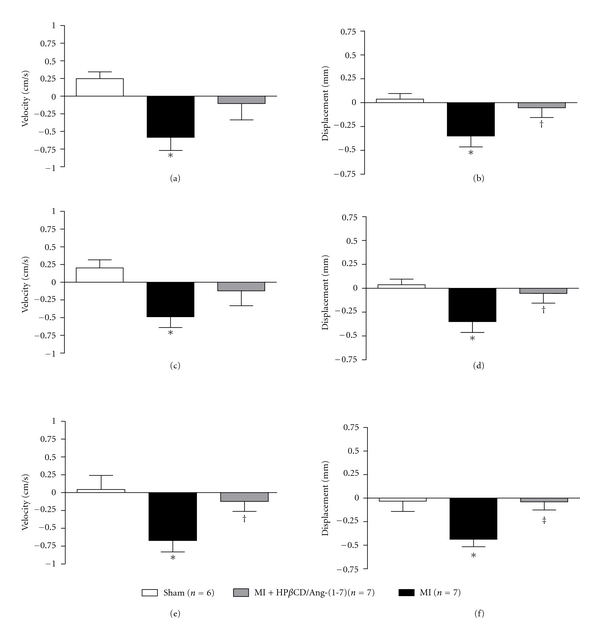
Effects of HP*β*CD/Ang-(1–7) on the variation of the velocity and displacement in rats after (a and b) 15 days; (c and d) 30 days, and (e and f) 50 days of treatment. *P < 0.05 versus sham; ^†^ *P* < 0.05 versus MI; ^‡^ *P* < 0.01 versus MI (one-way ANOVA followed by the Newman-Keuls posttest).

**Figure 4:**
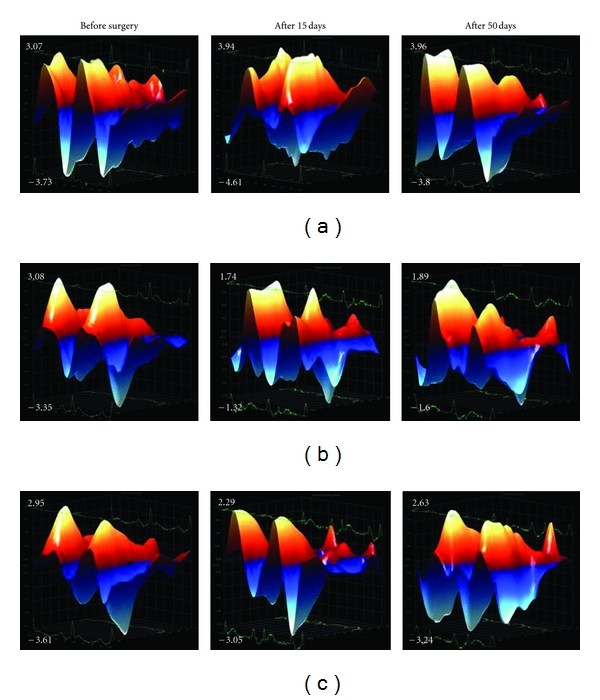
Representative images of the radial strain analysis of the velocity (cm/s) at basal conditions and after 15 and 50 days of MI. (a) sham group, (b) MI group, and (c) MI + HP*β*CD/Ang-(1–7).

**Figure 5:**
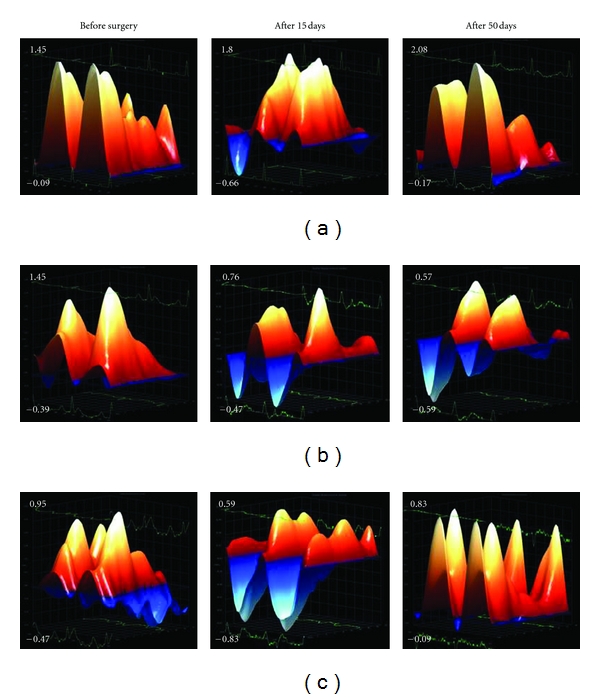
Representative images of radial strain analysis of the displacement (mm) at basal conditions and after 15 and 50 days of MI. (a) sham group, (b) MI group, and (c) MI + HP*β*CD/Ang-(1–7).

Radial strain analysis of the bidimensional long axis view of the LV revealed that MI induced a significant decrease in the velocity and displacement of myocardial fibers at 15, 30, and 50 days after surgery. HP*β*CD/Ang-(1–7) treatment completely reversed the reduction observed in the displacement of the myocardial fibers at all periods of evaluation after infarction (15, 30, and 50 days after surgery—Figures 3(b), 3(d), and 3(f), resp.) as well as in the velocity of myocardial fibers at 50 days after MI induction (Figure 3(e)). In addition, the velocity of myocardial fibers was improved in infarcted rats treated with HP*β*CD/Ang-(1–7) after 15 and 30 days of MI induction (Figures 3(a) and 3(b), resp.). Three-dimensional representative diagrams of velocity and displacement obtained by radial strain are shown in Figures 4 and 5, respectively.

### 3.4. Effects of HP*β*CD/Ang-(1–7) Long-Term Administration on Mas, TGF-*β*, and Collagen Type I mRNA Expression

**Figure 6:**
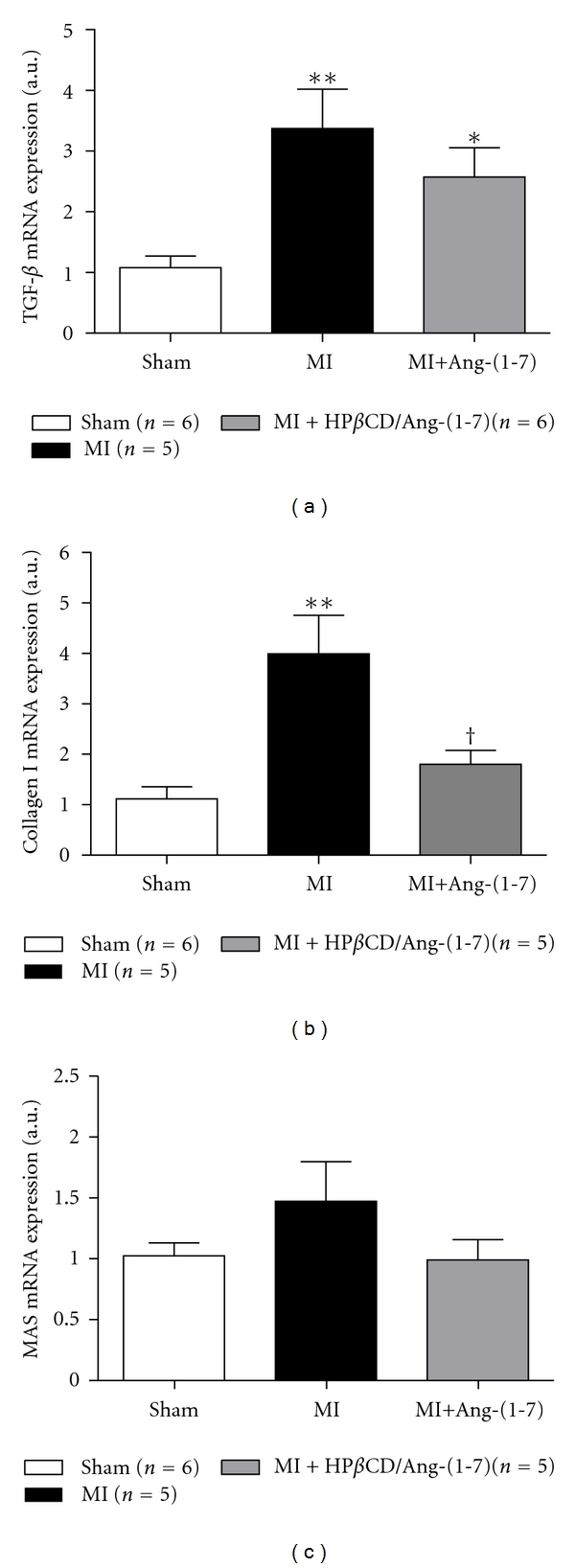
Effects of long-term administration of HP*β*CD/Ang-(1–7) on mRNA expression of (a) TGF-*β*, (b) collagen type I, and (c) Mas in infarcted animals. *P < 0.05 and **P < 0.01 versus sham; ^†^ *P* < 0.01 versus MI (one-way ANOVA followed by the Newman-Keuls posttest) (a.u) = Arbitrary Units.

At the end of the treatment, real-time PCR assays were performed in order to evaluate the Mas, TGF-*β*, and collagen type I gene expression. We found that vehicle-treated infarcted animals presented an increased expression of TGF-*β* (Figure 6(a)) and collagen type I (Figure 6(b)) in the heart as compared with sham-operated rats. The administration of HP*β*CD/Ang-(1–7) in MI animals abolished the increase of collagen type I mRNA expression and reduced the increase in the expression of TGF-*β* mRNA. Although the expression of Mas tended to increase in MI rats, it did not reach statistical significance when compared with sham-operated animals (Figure 6(c)). 

## 4. Discussion

In the present study, we demonstrated that once-a-day chronic oral administration of the inclusion compound HP*β*CD/Ang-(1–7) produced progressive time-dependent cardioprotective effects in MI animals. Specifically, we found that chronic oral administration of HP*β*CD/Ang-(1–7) improves the diastolic and systolic function and reduces the expression of fibrosis scar markers (TGF-*β* and collagen type I). 

MI is a common cause of heart failure in humans [2, 34, 35], and the rat model of MI produced by coronary artery ligation has been used extensively to study the pathophysiology of this condition as well as new approaches to its treatment [12, 35, 36]. In keeping with a previous study [37], our results indicate that the progressive increase in diastolic LV volume was the main mechanism underlying the maintenance of the stroke volume in the presence of a prominent decrease in the FS. Importantly, the treatment with HP*β*CD/Ang-(1–7) caused a significant time-dependent improvement in LV dilation demonstrated by the attenuation of the changes in ESLVV and EDLVV. 

It has been recently proposed that measurement of the myocardial deformation (velocity, displacement, strain, and strain rate) is a powerful technique to measure heart function disturbances [38, 39]. In this study, we used this approach to evaluate functional changes induced by MI and the effects of HP*β*CD/Ang-(1–7) on these alterations. A significant improvement in the velocity and displacement of the cardiac fibers in animals treated with this heptapeptide was observed.

The increased deposition of collagen in the heart and abnormal extracellular matrix structure result in myocardial stiffness, leading to ventricular systolic and diastolic dysfunction [24, 35, 37]. Our data are in keeping with these concepts since we observed that chronic treatment with HP*β*CD/Ang-(1–7) attenuated these alterations, evidenced by a smaller fall in EF and FS and by a smaller reduction in the thickness of the cardiac fibers in both systole (IVSDS) and diastole (IVSDD), which was accompanied by a decrease expression of TGF-*β* and collagen type I.

Cardiac fibroblasts are the primary source of TGF-*β* in the heart [30], and it was demonstrated that this cytokine is absolutely required for the Ang II-induced cardiac hypertrophy *in vivo* [30], and regulates the collagen synthesis in cardiac fibroblasts [40, 41]. Thus, there are evidences supporting a direct functional association between the RAS and TGF-*β* pathways [42]. It has been suggested that Smad proteins are the main downstream mediators of the cardiac Ang II/TGF-*β*1 pathway in the chronic phase of MI [28]. Furthermore, it was observed that TGF-*β*1/TAK1/p38MAPK-signaling pathway is activated in spared cardiomyocytes following MI and can play an important role in the development of hypertrophy in the remodeling myocardium [43]. On the other hand, it is well documented that Ang-(1–7) acting through the Mas receptor counter regulates the Ang II effects [4, 9, 10, 14, 18, 19, 44, 45]. Although our results did not show statistical differences in the expression of Mas among any of the groups, they clearly demonstrated that the administration of HP*β*CD/Ang-(1–7) in infarcted animals induced a reduction in the increase of TGF-*β* mRNA expression. These findings can explain, at least partially, the beneficial effects of the inclusion compound in MI. Moreover, the improvement in the heart function of MI rats by HP*β*CD/Ang-(1–7) treatment could be related to the reduction of the infarcted area as demonstrated in our previous study [12] which is in keeping with the strain analysis showing an improvement of the ventricular wall displacement in the HP*β*CD/Ang-(1–7)-treated MI rats.

One special feature of the Ang-(1–7) is its long-term effectiveness, as demonstrated in this study. Indeed, this observation is in keeping with previous studies showing beneficial effects of chronic Ang-(1–7) administration in different models of cardiovascular diseases [4, 11, 13, 14, 20, 44, 46]. Chronic Ang-(1–7) administration improved LV function of Wistar rats [47] and of diabetic spontaneously hypertensive rats (SHRs) [14] after global ischemia, attenuated the heart failure induced by MI [11], prevented the development of severe hypertension and end-organ damage in SHR treated with L-NAME [13], and reduced the cardiac remodeling in DOCA-salt and in Ang II-infused rats [4, 20]. In addition, an antifibrotic effect was observed in transgenic animals, which chronically present an increased plasma Ang-(1–7) levels [46].

In summary, this study showed that long-term treatment with HP*β*CD/Ang-(1–7) was able to attenuate the maladaptive remodeling events caused by MI, thereby indicating that Ang-(1–7) holds beneficial effects in hearts and that this inclusion compound constitutes an effective strategy to orally administer this heptapeptide.

## Figures and Tables

**Table 1:** Functional parameters during long term of continuous treatment with HP*β*CD/Ang-(1–7) in infarcted rats.

			Sham				MI			MI + HP*β*CD/Ang-(1–7)		
Parameters	Before	Days after surgery			Before	Days after surgery			Before	Days after surgery		
	surgery	15	30	50	surgery	15	30	50	surgery	15	30	50
Body weight (g)	188 ± 6	264 ± 6	342 ± 12	404 ± 11	191 ± 12	243 ± 11	307 ± 12	351 ± 14	213 ± 14	272 ± 10	334 ± 9	380 ± 11
LV mass (mg)	531 ± 39	695 ± 29	740 ± 34	793 ± 44	503 ± 31	740 ± 44	715 ± 26	901 ± 62	479 ± 54	640 ± 43	747 ± 46	780 ± 60
SV (*μ*L)	147 ± 9.6	179 ± 10	173 ± 7.8	181 ± 7.8	148 ± 10	147 ± 10.3	141 ± 13.8	163 ± 9.5	133 ± 12	170 ± 8.5	166 ± 8.9	175 ± 11
HR (bpm)	399 ± 17	395 ± 11	403 ± 16	380 ± 12	377 ± 10	367 ± 23	359 ± 10	351 ± 11	410 ± 6	366 ± 10	378 ± 11	368 ± 11
CO (mL/min)	58 ± 4	70 ± 4	70 ± 4	69 ± 4	56 ± 4	55 ± 7	51 ± 6	57 ± 3	54 ± 4	61 ± 3	63 ± 4	65 ± 5

Data are expressed as mean ± SEM. No significant differences were observed among any of the groups (two-way ANOVA followed by Bonferroni posttest). MI: myocardial infarction; LV: left ventricle; SV: stroke volume; HR: heart rate; CO: cardiac output.
